# Utility of Infrared Thermography in Dogs with Osteoarthritis

**DOI:** 10.3390/ani16152359

**Published:** 2026-08-02

**Authors:** Melissa J. Lewis, Elizabeth Kawecki-Wright, Amber L. Hall, Nathan P. Hall, Yang Qu, Masataka Enomoto, B. Duncan X. Lascelles

**Affiliations:** 1 Department of Clinical Sciences, College of Veterinary Medicine, North Carolina State University, Raleigh, NC 27607, USA; 2 Translational Research in Pain, College of Veterinary Medicine, North Carolina State University, Raleigh, NC 27607, USAalhall6@ncsu.edu (A.L.H.); nphall@ncsu.edu (N.P.H.); menomot@ncsu.edu (M.E.); dxlascel@ncsu.edu (B.D.X.L.); 3 Clinical Studies Core, Office of Research, College of Veterinary Medicine, North Carolina State University, Raleigh, NC 27607, USA; 4 Comparative Pain Research and Education Centre, College of Veterinary Medicine, North Carolina State University, Raleigh, NC 27607, USA; 5 Department of Anesthesiology, Center for Translational Pain Research, Duke University, Durham, NC 27710, USA; 6 Thurston Arthritis Center, University of North Carolina, Chapel Hill, NC 27599, USA

**Keywords:** thermal imaging, osteoarthritis, canine, pain, joint

## Abstract

Objective, noninvasive biomarkers of joint inflammation or pain are needed for dogs with osteoarthritis (OA). This prospective, observational study investigated whether body surface temperature quantified by infrared thermography was associated with joint pain and/or radiographic osteoarthritis (rOA) in dogs. Using thermal images, 14 regions of interest (ROI) were created for bilateral limb joints and the vertebral column for each dog and the mean temperature was calculated for each ROI. Orthopedic examination and radiographs classified each joint/region as affected or unaffected by rOA, pain, or both, called symptomatic OA (sxOA). Mean temperatures were compared between affected and unaffected joints. Eighteen dogs were enrolled, encompassing analysis of 250 ROI (rOA = 83/250, 33%; pain = 83/250, 33%; sxOA = 61/250, 24%). No temperature differences were detected between affected and unaffected joints for rOA (0.405 °C, 95% CI [−0.204, 1.025 °C]), pain (0.208 °C, 95% CI [−0.390, 0.806 °C]), or sxOA (0.276 °C, 95% CI [−0.344, 0.915 °C]). Elbow and stifle regions were warmer, and the lumbosacral region was cooler than the carpus. Long-haired dogs were cooler than short-coated dogs. Regional temperature variability and haircoat type might hinder detection of OA-related thermal signals. In dogs with OA, findings do not support infrared thermography as a standalone tool to identify affected joints.

## Simple Summary

Osteoarthritis is common in dogs, and simple, objective ways to reliably detect affected joints are needed. Using a thermal camera that captures body surface temperature, this study explored whether thermal imaging could detect osteoarthritic joints in dogs with one or more joints affected by mild-to-moderate disease. Thermal images were acquired in a series of 18 dogs, and the average surface temperature was calculated within regions of interest drawn over joints of the limbs and spine. Orthopedic examination and X-rays were used to classify each joint as affected or unaffected by osteoarthritis and compared to the temperature data. No temperature differences were detected between affected and unaffected joints across dogs. Elbow and knee regions were warmer, and the lower back region was cooler than the wrist. Long-haired dogs were cooler than short-coated dogs. Thermal imaging was unable to distinguish which joints were affected by osteoarthritis, in part, because surface temperature varies by region of the body and haircoat type. In dogs with osteoarthritis, findings do not support thermal imaging as a standalone tool to identify affected joints.

## 1. Introduction

Medical Infrared Thermography (IRT) is a noninvasive imaging modality used to measure radiant heat emitted in the electromagnetic spectrum [1,2]. In living organisms, this technology allows for the evaluation of changes in the body surface temperature (BST) due to local alterations in blood flow, which can be caused by a variety of physiologic or pathologic processes such as pain or inflammation [3]. Compared to other imaging modalities, IRT is quick, noninvasive, and does not require anesthesia or sedation. However, various patient characteristics (e.g., haircoat type), environmental (e.g., ambient temperature) and technical factors (e.g., imaging acquisition protocols) can impact IRT [4,5,6,7,8,9,10,11,12]. These sources of variability among individuals and testing conditions can interfere with interpretation and present unique challenges for clinical usage [4,5,6,7,8,9,10,11,12].

Osteoarthritis (OA) is the most common joint condition affecting both dogs and people and is associated with joint dysfunction, pain, decreased mobility, and negative impacts on other domains such as sleep and cognitive function [13,14,15,16,17,18]. In a cohort of dogs with no prior diagnosis of OA undergoing routine dental prophylaxis, 60% had radiographic evidence of OA (rOA), and 89% of those dogs had more than one joint affected [19]. Prevalence of OA-associated pain in dogs is 20–40% [15,20,21]. While the severity of OA-associated pain is variable, a substantial proportion of dogs demonstrate at least mild pain in a radiographically affected joint, referred to as symptomatic OA (sxOA) [19]. Screening for OA typically involves comprehensive physical and orthopedic examinations, as well as orthogonal radiographs of the appendicular and axial skeleton [15]. However, radiographic investigation is commonly performed only after a clinical suspicion of pain or decreased mobility is perceived, focusing primarily on the affected area, leading to potential under-recognition of affected animals [19]. These findings indicate that OA in dogs is currently underdiagnosed and undertreated. Therefore, an objective assessment tool that can screen for the presence and/or document the specific locations of OA would help veterinarians.

Thermography has been utilized in people to detect early stages of rOA as well as to assess response to therapeutic treatment [22,23,24,25]. In dogs, it has been used to evaluate individuals with unilateral pelvic limb lameness, cranial cruciate ligament-deficient stifles, elbow dysplasia, and coxofemoral OA [4,26,27,28,29,30,31]. However, the majority of these studies compared groups of clearly affected and normal dogs at single joint sites [26,27,28,30,31]. Among dogs with suspected or confirmed OA, the utility of IRT to identify early OA or differentiate which joints are affected by OA is not well explored, and its potential role as a screening or monitoring tool in this population has yet to be established or validated.

The purpose of this pilot study was to investigate whether BST as quantified by IRT was associated with individual joint pain and/or radiographic joint arthritis in a cohort of dogs diagnosed with rOA affecting at least one joint. We hypothesized that IRT would be able to distinguish between affected and unaffected joints and, in particular, identify dogs with sxOA.

## 2. Materials and Methods

### 2.1. Patient Selection

Dogs were recruited from a pre-existing, prospective observational clinical study investigating the prevalence of OA conducted by the Translational Research in Pain Program (TRiP) at NCSU College of Veterinary Medicine. Dogs ≥ 5 years old of any breed or sex status weighing at least 10 kg were eligible for evaluation if they were systemically healthy enough to undergo sedation. The enrollment period for the parent study was from August 2024 to October 2025. That study protocol is outlined elsewhere [32], but dogs for this pilot were enrolled in either the initial screening arm or the 8-month longitudinal follow-up arm of the parent study. Dogs with underlying neurological disease or disease processes that affected gait or mobility were excluded. Dogs were eligible for inclusion in the pilot IRT study if they had evidence of rOA in at least one appendicular region or axial joint confirmed by orthogonal view radiographs. For the purposes of this study, rOA encompassed degenerative joint changes in a synovial joint or of the vertebral column. All procedures for the parent and pilot studies were approved by the NC State IACUC (protocol #22-245- O and 24-162, respectively) and all owners provided informed consent. Methodology and results for this work were reported in compliance with the STROBE-Vet and PetSORT guidelines [33,34].

### 2.2. Patient and Radiographic Evaluation

For each participant, recorded variables included age, breed, sex status, body condition score, characterization of haircoat type (subjectively classified as either long or short), and current medications or supplements.

Orthopedic examination consisted of evaluation of signs of pain for each appendicular joint (mani, carpi, elbows, shoulders, pes, tarsi, stifles, hips) and each part of the axial skeleton (cervical, thoracic, thoracolumbar junction, lumbar, and lumbosacral regions). Each dog was assessed by one of two evaluators (EKW, ME) with 5 and >15 years of experience, respectively, performing orthopedic examinations and diagnosing OA. Pain associated with OA was graded on the previously described scale of 0–4 [35], where a score of 0 indicates no identifiable pain, 1 is defined as orients to site on manipulation, does not resist or only mild resistance, 2 is defined as orients to site, slight objection to manipulation, 3 is defined as withdraws from manipulation, may vocalize, may turn to guard area and 4 indicates tries to escape from manipulation, or prevent manipulation, may bite or show aggression on manipulation. For analysis purposes, joint pain was classified as affected (score of 1–4) or unaffected (score of 0) for each joint. To avoid radiographic findings influencing assessment of pain, orthopedic examination and pain scoring were performed prior to obtaining and reviewing radiographs.

Orthogonal radiographs were performed using a standardized sedation protocol (mu-opiate and an alpha-2 adrenergic agonist, adapted to the needs of the individual dog) of the following joints: mani, carpi, elbows, shoulders, pes, tarsi, stifles, and hips as well as the entire vertebral column. Images were assessed independently for rOA by two trained veterinary evaluators (EKW and ME). A subjective rOA rating scale was used to assign a number to each joint using a previously described technique, where 0 = no radiographic abnormalities identified and 10 = the most severe radiographic changes associated with OA [36]. Radiologic features considered indicative of the presence of appendicular OA were osteophytes, enthesophytes, sclerosis, subchondral bone erosions and cysts. The rOA changes for each axial segment were assessed using the same numerical scale described above (0 = no radiographic abnormalities identified, and 10 = ankylosis). Radiographic features evaluated and considered indicative of degenerative changes in the axial skeleton were osteophytes, spondylosis, disk-associated degeneration (end plate sclerosis, erosion, disk mineralization, narrowing), and subluxation. The vertebral column was divided into two regions (thoracolumbar and lumbosacral), and any rOA changes within a region were considered together (rather than attempting to utilize individual vertebral levels to avoid over-emphasizing overlapping changes within adjacent levels that cannot be clearly distinguished clinically). Each joint/region was recorded as binary ‘affected (score of 1–10)/unaffected (score of 0);’ any discrepancies were discussed until consensus was reached.

Dogs were classified as having sxOA if both rOA and joint pain were present for a given joint. For analysis purposes, the classification of rOA, pain and sxOA for each dog was based on a single parent study visit that occurred prior to the IRT evaluation visit and the time interval between these two visits was recorded. Pain assessments (i.e., orthopedic examinations) and rOA assessments (i.e., radiographs) were variably repeated at other visits as part of the parent study, allowing determination of stability over time regarding pain and rOA status.

### 2.3. Thermal Imaging Acquisition

Thermographic images were captured using the WellVu 640 (Wellvu Veterinary Thermal Imaging, Solon, OH, USA). Thermal camera specifications include a medically calibrated lens (approximately 200-degree thermal window), 640 × 512 pixel array, 0.02 °C sensitivity and an emissivity setting of 1. To minimize environmental variability, imaging was performed in a dedicated room with the temperature maintained at 20 °C in an area of low air movement and indirect lighting. All dogs had a minimum 10 min acclimation period with no handling. Wearable accessories (collars, leashes, clothing) were removed prior to the start of the acclimation period. Handlers wore two sets of nitrile examination gloves to reduce potential thermal interference and avoided direct physical contact with body areas to be imaged. Slip-leads were utilized if needed to guide subjects through the image series.

Dogs were positioned standing squarely in front of a black matte background while standing on a dark-colored yoga mat. The camera was held with the lens pointed at a perpendicular angle to the area of the body being imaged and with the dog centered and filling the majority of the screen. Left and right lateral, dorsal, cranial and caudal images were captured for each dog in grayscale with the fur in clear focus.

### 2.4. Imaging Analysis

Adapted from Loughin et al., 14 regions of interest (ROI) were defined [9]. This included left and right carpus, elbow, shoulder, tarsus, stifle, and hip and thoracolumbar and lumbosacral spine. The cervical region, manus and pes were excluded from analysis due to variable head positioning or inclusion of the feet between dogs; no dogs included in the pilot study had OA exclusively localized to the cervical spine or feet.

**Figure 1:**
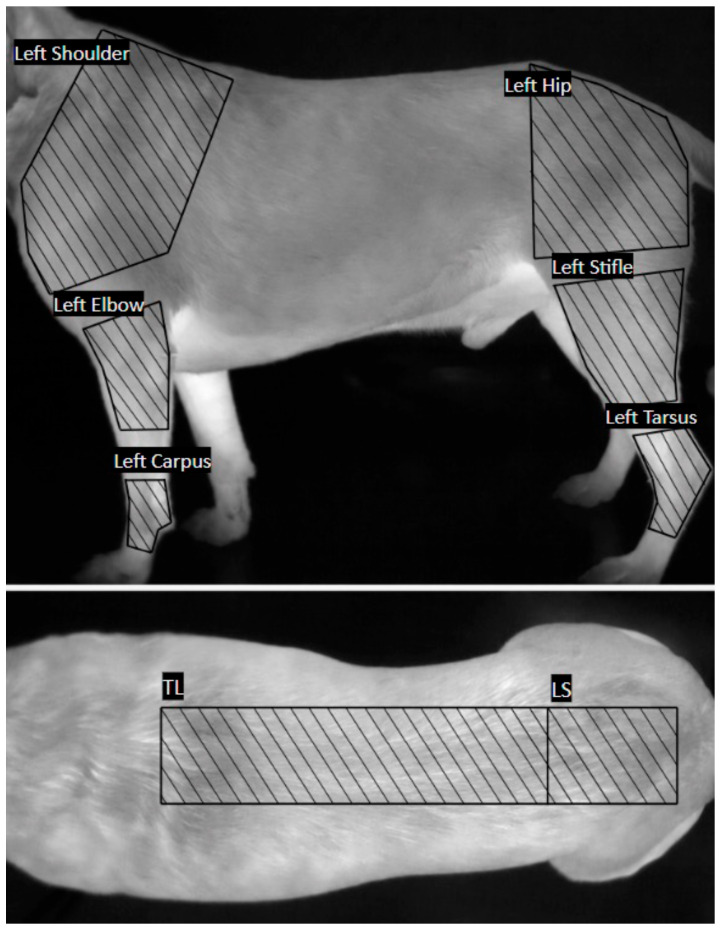
Representative depiction of regions of interest manually drawn on thermal images.

Using lateral and dorsal images in grayscale mode, the ROI were manually drawn by two authors (AH, NH) without knowledge of patient clinical or radiographic details (Figure 1). Each joint ROI consisted of a polygon centered over the joint of interest, extending just proximal and distal to the joint, with the contours of the limb defining the cranial/dorsal and caudal/palmar/plantar borders. The thoracolumbar ROI was centered over midline and extended from where the thoracic limbs met the body to where the pelvic limbs met the trunk. The lumbosacral ROI was also centered over the midline and extended from where the cranial aspect of the pelvic limbs met the body to the start of the tail head. Any shaved areas were excluded from the ROI, or if that was not possible, then that joint was excluded from analysis. For each ROI in each dog, the WellVu IR Pro software version 3.4.5 calculated the mean temperature.

### 2.5. Statistical Analysis

Analyses were performed using commercially available statistical software (Microsoft Excel Version 2511, SPSS 29.0.1, and R version 4.5.1). As a pilot study with inherent limitations for power analysis and sample size estimation, all results should be considered exploratory. Demographic data and various characteristics of the study population were summarized using descriptive statistics, including calculating proportions and percentages, mean and standard deviation or median and range, as appropriate. We used the tidyverse R package to compute the summary statistics, and ggplot2 to visualize the data.

For each OA outcome (rOA, joint pain, and sxOA), the averaged BST value was compared between affected and unaffected joints or regions using a nonparametric bootstrap resampling approach on a pooled sample (i.e., pooling all regional measurements for an individual dog). The arithmetic mean of the bootstrapped differences between affected and unaffected joints served as the point estimate, while the 2.5th–97.5th percentiles defined the 95% confidence interval (CI). If the interval included zero, the difference was considered non-significant. For all three primary variables, Cohen’s d effect sizes were calculated based on the bootstrap estimates and interpreted using standard thresholds: values less than 0.2 were considered small, values around 0.5 indicated a medium, and values greater than 0.8 indicated a large effect [37].

To evaluate the association of BST with both OA status and body region, a linear mixed-effects model was utilized, with individual dogs included as a random factor. For the fixed body region effect, the left carpus was set as the reference level. In addition to these primary fixed effects, dog age, sex, body condition score, and haircoat type were included as covariates. To maximize statistical power, secondary univariate analyses were conducted. These analyses evaluated the relationship between BST and each covariate, stratified separately by ROI. We used the lme4 package to fit our mixed model, lmerTest to get *p*-values for fixed effects, and emmeans to compute the estimated marginal means and pairwise comparisons. All *p*-values from the secondary univariate analyses were adjusted for multiple comparisons using the Benjamini–Hochberg procedure [38] with a standard significance threshold of *p* < 0.05 and an exploratory threshold of *p* < 0.1.

To further assess how haircoat type influenced the association between BST and OA status, a bootstrap procedure was performed on the ROI pooled sample for each haircoat type and Cohen’s d was calculated to estimate effect sizes.

## 3. Results

Twenty-three dogs were initially enrolled in the pilot study. Five dogs were excluded due to insufficient image acquisition, leaving a total of 18 dogs. Due to dog recruitment occurring at various points along the parent study timeline, the time between evaluation of OA status (rOA, joint pain and sxOA) and thermographic image acquisition was variable. The median number of days between data collection was 14 days (range 6–225 days), with 16 (89%) dogs having an interval of less than 3 weeks. For the two dogs with a longer interval, rOA assessments did not change in the approximately 6 months between radiographs (with the IRT visit in between). However, pain scores for five joints/regions in these two dogs differed between the initial (where OA status was classified) and IRT visits. For this reason, analyses were performed with and then without including these two dogs.

The mean age of enrolled dogs was 9 years (SD ± 1.7). Mean body weight was 29.1 kg (SD ± 8.8). Twelve dogs (67%) were male (11 neutered, one intact) and six (33%) were female (all spayed). Mixed breed dogs were the most reported breed. Median body condition score was 5 (range 4–8). Thirteen (72%) dogs had short haircoats and five (28%) dogs had long haircoats. Five dogs were on analgesic-type medications at the time of participation in this study, including gabapentin in three dogs, amantadine in one dog, bedinvetmab (<1 month since injection) in one dog, oromucosal dexmedetomidine as needed and carprofen in one dog. Two dogs were on supplements including omega-3 fish oil in one dog and Dasuquin, omega-3 fish oil, cobalequin, a probiotic and an immune support supplement in one dog. Administration of these medications and supplements was the same at the time of OA assessment and thermal imaging, except the one dog on carprofen was not on carprofen at the OA assessment visit. Details of individual patient characteristics are outlined in Table S1.

Across the 14 ROI in 18 dogs (252 ROI total), two carpal ROI from one dog were excluded due to unavoidable shaved spots from prior venipuncture, leaving 250 ROI for analysis. The distributions of rOA, joint pain and sxOA are outlined in Table 1. Eighty-three of 250 (33%) joints had evidence of rOA; rOA was noted in one joint in two dogs; two joints in two dogs; 3–4 joints in 10 dogs, and >5 joints in four dogs. The most common rOA locations were the thoracolumbar spine and hips. Eighty-three of 250 (33%) joints were assessed as painful, and the median pain score across painful joints was 1 (range 1–3), compatible with mild-to-moderate pain status as a group. The vertebral column and hips were the regions most commonly assessed as painful. Sixty-one of 250 (24%) joints were assessed as having sxOA where there was concurrent pain and rOA. Symptomatic OA was most common for the vertebral column, hips and right stifle. The least affected joints by sxOA were the tarsus, carpus and right shoulder.

Summary and distribution of temperature data for each OA status (rOA, joint pain, sxOA) are presented in Table 2. In the pooled analysis, no significant mean BST differences were identified between joints with or without rOA (0.405 °C, 95% CI −0.204, 1.025 °C), joint pain (0.208 °C, 95% CI −0.390, 0.806 °C), and sxOA (0.276 °C, 95% CI −0.344, 0.915 °C). Effect size estimates for each of the three OA categories were small (rOA d = 0.168, joint pain d = 0.087, sxOA d = 0.116). The linear mixed model also showed no statistically significant association between BST and rOA (*p* = 0.87), joint pain (*p* = 0.93), or sxOA (*p* = 0.39) status. After removal of the two dogs with a longer interval between OA assessment and thermal imaging, findings were comparable, with no significant mean BST differences identified for rOA (0.263 °C, 95% CI −0.323, 0.841 °C), pain (0.317 °C, −0.256, 0.890) and sxOA (0.260 °C, 95% CI −0.349, 0.869 °C).

**Figure 2:**
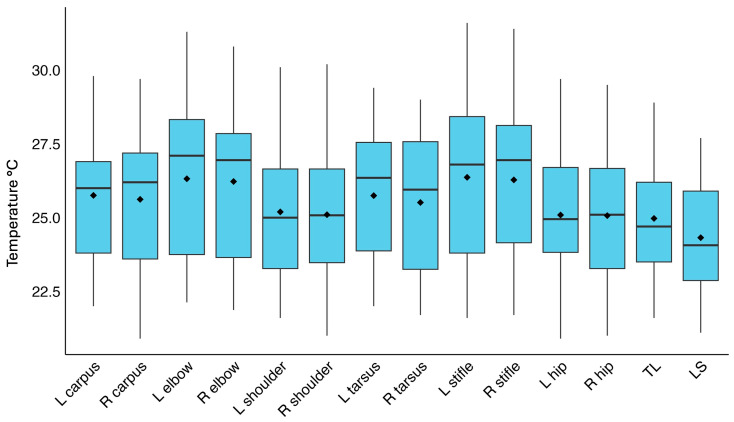
Boxplots of mean BST for each ROI. Each box depicts the mean (point), median (middle line), 25th (bottom) and 75th (top) quartiles with whiskers representing the farthest data points still within 1.5 times the interquartile range. BST: body surface temperature, ROI: region of interest; L: left; R: right; TL: thoracolumbar region; LS: lumbosacral region.

Summary and distribution of temperature data for each ROI are presented in Table 3 and Figure 2. Compared to the reference region (designated as the left carpus) and accounting for OA status, the left and right elbow and left and right stifle regions exhibited significantly higher mean BST (*p* = 0.02, 0.03, 0.01, 0.02, respectively), and the lumbosacral region showed a significantly lower mean BST (*p* < 0.001). After inclusion of additional covariates in the model (sex, age, body condition score and haircoat type) and adjusting for multiple comparisons, body region remained a significant predictor of temperature variation for the elbow and stifle regions. No significant effect was identified for OA status or the other covariates (sex, age, body condition score and haircoat type), suggesting these factors did not independently contribute to BST differences in this dataset. However, combined mean BST across ROI was higher for dogs with short haircoats (26.15 °C, SD 2.44 °C) than those with long haircoats (23.97 °C, SD 1.55 °C). Additionally, univariate analysis of each covariate, stratified by body region, identified that haircoat type was possibly associated (using our exploratory threshold after adjustment of *p* < 0.1) with mean BST for eight ROI: left and right carpus (adjusted *p* = 0.06, 0.08, respectively), elbow (adjusted *p* = 0.08, 0.06, respectively), tarsus (adjusted *p* = 0.06, 0.08, respectively), and stifle (adjusted *p* = 0.08, 0.06, respectively), suggesting a potential influence of haircoat type, at least for certain areas. The aforementioned relationships were comparable after removal of the two dogs with longer intervals between OA assessments and thermal imaging.

Summary and distribution of temperature data for each OA status, separated by haircoat type, are outlined in Table 4. Among the 70 ROI in the five long-haired dogs, 19/70 (27%) were affected by rOA and 51/70 (73%) were unaffected by rOA (Table 5); one dog had 11/14 ROI with sxOA while the remaining four dogs had 0-2/14 ROI with sxOA. Among the 180 ROI in the 13 short-haired dogs, 64/180 (36%) were affected by rOA and 116/180 (64%) were unaffected by rOA; the number of ROI with sxOA per dog ranged from 1 to 11. Considering just the long haircoat group, there were significant positive mean temperature differences (i.e., higher BST) for joints with rOA (2.142 °C, 95% CI 1.300, 2.850 °C), joint pain (2.225 °C, 95% CI 1.300, 2.950 °C), and sxOA (2.185 °C, 1.200, 3.100 °C) compared to those without rOA, pain and sxOA. Effect size estimates for each of the three OA categories were large (rOA d = 1.533, joint pain d = 1.576 and sxOA d = 1.485). No significant mean temperature differences were identified for the short hair group between joints with or without rOA (−0.337 °C, 95% CI −1.061, 0.389), joint pain (−0.484 °C, 95% CI −1.196, 0.226), and sxOA (−0.451 °C, −1.235, 0.340) and effect sizes were small (rOA d = −0.137, joint pain d = −0.204, sxOA d = −0.188). This was unchanged after removing the two dogs, both in the short hair group, with longer intervals between OA assessments and thermal imaging.

## 4. Discussion

In a group of dogs diagnosed with OA affecting at least one joint, BST quantified by IRT was unable to identify joints or regions with rOA, pain, or sxOA, thus rejecting our hypothesis. However, body region and haircoat type appeared to influence temperature values, which might have impacted the ability to broadly detect joint changes. Taken together, these results suggest a limited role for IRT as a standalone tool for determining which joints are affected by OA, especially in less severely affected dogs.

IRT can indirectly detect inflammation through localized changes in BST [3]. Osteoarthritis is a progressive, degenerative process which can lead to destruction of joint cartilage, osteophyte formation, joint instability, and other inflammatory changes [15]. Since OA results in joint inflammation and frequently affects multiple joints to varying degrees within a given dog [21,32], we postulated that IRT might be useful as a simple, noninvasive tool to identify OA in dogs. Prior studies in people support that IRT can identify early OA in some joints, particularly in the knee and hand [22,23,24,25]. Similarly, IRT in dogs has been used to distinguish between normal and affected joints across several conditions, including elbow and hip dysplasia and cranial cruciate ligament deficiency [26,27,28,29,30]. However, those studies where significant temperature differences were identified focused on single joint comparisons in dogs where the abnormal status was clinically obvious [26,27,28], and its use as a broad tool across joints in dogs with OA has been minimally explored. In our cohort of dogs, IRT was unable to differentiate between affected and unaffected joints. This discrepancy with some prior studies might be attributed to differences in the severity of inflammation and clinical signs seen with various causes of joint disease. Osteoarthritis is associated with chronic, mild inflammation, while conditions such as acute cranial cruciate ligament rupture are typically associated with more pronounced effusion, pain and acute inflammation [39,40,41]. It is possible that the nature of OA-associated inflammation (in the absence of a clinically apparent exacerbation) does not result in consistent alterations in overlying BST in dogs. Ultimately, the reasons are likely multifactorial, but findings suggest limited potential for IRT as a routine tool for determining which joints are affected by OA.

Within the context of OA-affected joints, we speculated that IRT would be most useful for joints that were both painful and radiographically arthritic (i.e., sxOA). However, IRT was globally unable to differentiate between affected or unaffected joints, regardless of whether that was rOA, pain or sxOA. This could be at least partially attributed to our study design, which categorized each joint as a binary for each status, either affected or unaffected. As such, the affected regions could vary from mild to more severe disease burden or pain. Among the regions designated as painful, the majority were scored as a ‘1 ’, which corresponds to mild demonstrable pain. The relatively milder OA disease burden in this cohort could have mitigated the ability of IRT to detect a difference between affected and unaffected joints. It also remains unclear how more severe OA changes impact thermographic BST, as lower temperature has been described in dogs with more severe hip OA compared to mild-to-moderate hip OA, while higher temperature patterns are described for dysplastic elbows [28,29,42]. Scoring of joint pain is also somewhat subjective; a small subset of the dogs received analgesic medications, and clinical signs of OA do not reliably correspond to the presence or severity of OA changes on radiographs [43,44,45]. This could have led to variable classification of affected status and impacted IRT results. Additionally, the severity of rOA changes or pain does not necessarily correlate with markers of joint inflammation [41,46,47], and lack of such relationships could limit the utility of IRT as a marker of disease. It remains challenging to differentiate between clinically apparent (i.e., sxOA) and clinically inapparent (i.e., rOA only) OA, especially in the context of a milder disease phenotype, and our results suggest that IRT is unlikely to be a simple fix for this challenging clinical scenario.

Multiple environmental and patient factors have been shown to influence BST quantified by IRT, which could further complicate interpretation of pathologic joints. Temperature variability between anatomic regions has been demonstrated with IRT in dogs with warmer regions in areas with thinner fur coverage or closer to core body structures [5,9,10]. Our results demonstrated that elbow and stifle ROI were significantly warmer and the lumbosacral region was cooler, relative to the carpus, and regardless of OA status. The elbow and stifle might be warmer than the carpus due to an even more peripheral location for the latter. The lumbosacral region might be cooler than the carpus due to regionally thicker haircoat and less vascularity. Significant variation in BST of the caudal lumbar region has been identified among different breeds of working dogs with hip OA, a finding possibly attributed at least in part to differences in coat thickness [4]. The decision to utilize the carpus as the reference joint might have impacted the relative changes identified, but regardless, this reinforces that there is normal regional BST variability in dogs. Since we grouped all affected and unaffected ROI, this might have masked the ability to detect OA-associated changes. It further suggests that inherent temperature variability across body regions might hinder the utility of IRT as a body-wide tool for detecting which areas are affected by OA, or at least that this needs to be considered in analysis models.

Haircoat type is another factor that has been demonstrated to impact BST in dogs. Short-haired dogs have warmer BST than long-haired dogs [7,9,10,11]. While including haircoat type as a covariate did not suggest this had an independent influence on temperature in this cohort, univariate analysis suggested that temperature might differ between short and long haircoats, particularly for certain areas of the body. Interestingly, among the ROI from just long-haired dogs, joints affected by rOA, pain and sxOA were significantly warmer than unaffected joints, while no significant differences were identified among the ROI from short-haired dogs. The reason for this finding is unclear. Among the short-haired dogs, a larger percentage of unaffected joints were located more distally (including elbow and stifle ROI) compared to affected joints. Since the elbow and stifle ROI tended to be warmer, this could have made the unaffected group appear warmer and limited the ability to detect a difference based on OA status. No clear distribution pattern was apparent for the long-haired group, and it is possible that the generally cooler BST in long-haired dogs allowed improved detection of temperature changes attributable to OA status. However, these results should be interpreted very cautiously as the long-haired group consisted of only five dogs and might have been driven by random variance or a single dog with a more severe burden of OA (11 out of 14 ROI affected). Our results reinforce that haircoat type is likely an important source of inter-individual BST variability. Similar to regional variability, haircoat needs to be considered in analysis models and complicates the use of IRT as a tool for determining OA status and extent.

A main limitation of this study is the small sample size in which there were non-independent observations (i.e., 250 observations analyzed from just 18 dogs). While we tried to account for this in our statistical methods by using a robust/conservative method in the bootstrap analysis, the sample size was inherently small; there are drawbacks to our pooled approach, and coupled with multiple comparisons in our analyses, results should be considered exploratory. While no overt relationships were identified for most of the covariates examined, the study cohort consisted of all older, relatively larger dogs with relatively uniform body condition scores, likely limiting the ability to detect an influence of these various factors. The study group generally had mild-to-moderate OA, based on the number of affected joints and average pain scores, which might also have limited detection of relationships. However, this represents a common and clinically relevant cohort for which IRT might not be as useful. Additionally, some individual dogs or joints were more severely affected, but OA severity was not considered, precluding determination of a relationship between BST and severity. Another limitation is that a small proportion of the dogs were administered supplements or analgesic medications during the study period. In addition to potential influence on OA classification, it is possible their administration influenced physiologic parameters like local blood flow or inflammation and could have impacted thermal imaging results. For logistical reasons, determination of OA status and IRT did not occur at a single visit, and the timing of OA assessments relative to when IRT was performed was not standardized, which could have impacted results. While this interval was short (less than a few weeks) for most dogs and their clinical status remained stable, minor dynamic variations in disease status, particularly in assessment of pain, could have resulted in the initial assessment of OA status being inaccurate for some ROI at the time of thermal imaging. Similarly, while results did not differ after removing the two dogs with longer intervals and their rOA status was unchanged during that time period, their disease status may still have changed over a period of months in ways that could have influenced our designations of disease status and subsequent comparisons. Inter- or intra-rater agreement was not assessed and might have further impacted the validity of our classification of OA status. We utilized a quantitative approach to compare mean BST between affected and unaffected regions to avoid over-interpretation of potential visual differences between joints. Since qualitative analysis using relative thermal (color) patterns is often used clinically, future work could compare quantitative and qualitative analyses, including investigation of mean, minimum and maximum values. We also utilized a minimum 10 min acclimation period before IRT, but there is no clear consensus on the necessary duration, and it is possible that a longer period would have been more appropriate. Our cohort only included dogs with confirmed OA in at least one joint without an unaffected control group for comparison and as such, the role of IRT as a true screening tool for initial OA diagnosis remains to be elucidated. We also only evaluated a single timepoint and cannot assess the value of IRT for serial monitoring over time in individual dogs with OA affecting one or multiple joints.

## 5. Conclusions

In this cohort of dogs with OA, IRT demonstrated an inability to differentiate joints by their OA status, whether that was radiographic changes, pain or a combination. Regional variability and haircoat type were factors influencing temperature data. Future work could explore targeted applications for IRT such as its utility for more severely affected dogs, serial monitoring or response to therapy.

## Figures and Tables

**Table 1:** Distribution of rOA, joint pain and sxOA for each ROI, presented as n/N (%). rOA: radiographic osteoarthritis; sxOA: symptomatic osteoarthritis; ROI: region of interest. * Data excluded for left and right carpus in one dog.

ROI	rOA	Joint Pain	sxOA
Left carpus *	3/17 (18%)	2/17 (12%)	2/17 (12%)
Right carpus *	3/17 (18%)	2/17 (12%)	2/17 (12%)
Left elbow	6/18 (33%)	4/18 (22%)	4/18 (22%)
Right elbow	5/18 (28%)	5/18 (28%)	4/18 (22%)
Left shoulder	6/18 (33%)	5/18 (28%)	4/18 (22%)
Right shoulder	4/18 (22%)	5/18 (28%)	2/18 (11%)
Left tarsus	6/18 (33%)	1/18 (6%)	1/18 (6%)
Right tarsus	5/18 (28%)	2/18 (11%)	2/18 (11%)
Left stifle	5/18 (28%)	4/18 (22%)	3/18 (17%)
Right stifle	6/18 (33%)	7/18 (39%)	6/18 (33%)
Left hip	8/18 (44%)	11/18 (61%)	8/18 (44%)
Right hip	8/18 (44%)	11/18 (61%)	8/18 (44%)
Thoracolumbar	12/18 (67%)	13/18 (72%)	9/18 (50%)
Lumbosacral	6/18 (33%)	11/18 (61%)	6/18 (33%)
Total/Combined regions	83/250 (33%)	83/250 (33%)	61/250 (24%)

**Table 2:** Summary and distribution of temperature data (in °C) stratified by each OA group. Values represent the combined mean of the mean temperatures as well as minimum and maximum mean temperatures for affected and unaffected joints for each OA status.

OA Group	Temperature (°C)		
	Mean (SD)	Minimum	Maximum
rOA			
- Affected (N = 83 ROI)	25.81 (2.20)	22.0	31.6
- Unaffected (N = 167 ROI)	25.41 (2.54)	20.9	31.3
Joint pain			
- Affected (N = 83 ROI)	25.68 (2.04)	22.1	31.4
- Unaffected (N = 167 ROI)	25.47 (2.60)	20.9	31.6
sxOA			
- Affected (N = 61 ROI)	25.75 (2.15)	22.1	31.4
- Unaffected (N = 189 ROI)	25.48 (2.52)	20.9	31.6

**Table 3:** Summary and distribution of temperature data (in °C) for each ROI. Values represent the combined mean of the mean temperatures as well as minimum and maximum mean temperatures for each ROI across the dogs.

ROI	Temperature (°C)		
	Mean (SD)	Minimum	Maximum
Left carpus	25.76 (2.36)	22.00	29.80
Right carpus	25.62 (2.67)	20.90	29.70
Left elbow	26.32 (2.78)	22.13	31.30
Right elbow	26.23 (2.70)	21.87	30.80
Left shoulder	25.20 (2.22)	21.60	30.10
Right shoulder	25.10 (2.20)	21.00	30.20
Left tarsus	25.75 (2.35)	22.00	29.40
Right tarsus	25.51 (2.47)	21.70	29.00
Left stifle	26.37 (2.84)	21.60	31.60
Right stifle	26.28 (2.87)	21.70	31.40
Left hip	25.09 (2.18)	20.90	29.70
Right hip	25.07 (2.24)	21.00	29.50
Thoracolumbar	24.97 (1.85)	21.60	28.90
Lumbosacral	24.32 (1.88)	21.10	27.70

**Table 4:** Summary and distribution of temperature data (in °C) for each OA group, stratified by haircoat type. Values represent the combined mean of the mean temperatures as well as minimum and maximum mean temperatures for affected and unaffected joints for each OA status, stratified by long or short haircoat type.

OA Group	Temperature (°C)		
	Mean (SD)	Minimum	Maximum
Long haircoat (N = 5 dogs, 70 ROI)			
rOA			
- Affected (N = 19 ROI)	25.40 (1.30)	22.71	28.10
- Unaffected (N = 51 ROI)	23.42 (1.27)	20.90	27.70
Joint pain			
- Affected (N = 24 ROI)	25.33 (1.55)	22.71	28.10
- Unaffected (N = 46 ROI)	23.28 (0.99)	20.90	25.40
sxOA			
- Affected (N = 15 ROI)	25.55 (1.39)	22.71	28.10
- Unaffected (N = 55 ROI)	23.56 (1.29)	20.90	27.70
Short haircoat (N = 13 dogs, 180 ROI)			
rOA			
- Affected (N = 64 ROI)	25.93 (2.40)	22.00	31.60
- Unaffected (N = 116 ROI)	26.27 (2.47)	20.90	31.30
Joint pain			
- Affected (N = 59 ROI)	25.82 (2.21)	22.10	31.40
- Unaffected (N = 121 ROI)	26.31 (2.54)	20.90	31.60
sxOA			
- Affected (N = 46 ROI)	25.81 (2.35)	22.10	31.40
- Unaffected (N = 134 ROI)	26.26 (2.47)	20.90	31.60

**Table 5:** Distribution of rOA status grouped by distal (carpus, tarsus, elbow, stifle) versus proximal (shoulder, hip, vertebral column) ROI and stratified by long or short haircoat type.

OA Group	Distal ROI	Proximal ROI
Long haircoat (N = 5 dogs, 70 ROI)		
rOA		
- Affected (19 ROI)	10/19 (53%)	9/19 (47%)
- Unaffected (51 ROI)	30/51 (59%)	21/51 (41%)
Short haircoat (N = 13 dogs, 180 ROI)		
rOA		
- Affected (64 ROI)	29/64 (45%)	35/64 (55%)
- Unaffected (116 ROI)	73/116 (63%)	43/116 (37%)

## Data Availability

The raw data supporting the conclusions of this article will be made available by the authors upon reasonable request.

## Supplementary Materials

The following supporting information can be downloaded at: https://www.mdpi.com/article/10.3390/ani16152359/s1, Table S1: Demographic and patient characteristics.
