# Supplementary material for: Utility of Infrared Thermography in Dogs with Osteoarthritis
Source: Animals (Basel). 2026 Aug 2;16(15):2359. doi: 10.3390/ani16152359 (PMC13463705; doi:10.3390/ani16152359)
Supplement: Supplementary file 1 [file animals-16-02359-s001.zip › animals-4437760-supplementary.pdf]

Supplemental Table S1. Demographic and patient characteristics.

Dog	Breed	Sex	Weight (kg)	BCS (x/9)	Age visit (years)	Hair coat type	Medications during the study period	Supplements during the study period
1	American Foxhound	FS	31.2	5	9.5	Short	gabapentin amantadine	glucosamine/chondroitin sulfate, omega-3 fatty acids, cobalamin/folate, probiotic, immune support supplement
2	English Setter	MN	25.1	5	9.9	Long	None	None
3	Mixed Breed Dog	MN	23.9	5	6.7	Short	dexmedetomidine PRN, gabapentin, trazodone	omega 3 fatty acid
4	Mixed Breed Dog	MN	30.0	6	9.5	Short	levetiracetam	None
5	Golden Retriever	FS	28.5	5	6.2	Long	None	None
6	Labrador	MN	36.4	6	6.4	Short	carprofen, fluoxetine	None
7	Mixed Breed Dog	FS	39.7	5	8.1	Short	None	None
8	Mixed Breed Dog	MN	41.2	4	10.5	Short	None	None
9	Mixed Breed Dog	MN	22.7	5	12.0	Short	None	None
10	Golden Retriever	MN	32.0	5	7.5	Long	None	None
11	Mixed Breed Dog	MN	28.2	4	8.3	Short	bedinvetmab	None
12	Greyhound	MN	29.5	4	11.1	Short	None	None
13	English Springer Spaniel	FS	20.0	6	9.7	Long	None	None

14	Beagle	FS	11.0	5	8.9	Short	None	None
15	Old English Sheepdog	MN	48.1	8	7.6	Long	None	None
16	American Pit Bull Terrier	FS	20.0	4	11.6	Short	gabapentin, cetirizine doxycycline	None
17	Boxer	MN	32.6	5	9.7	Short	None	None
18	Mixed Breed Dog	MI	22.8	5	9.2	Short	oclacitinib	None
